# The role of EMMPRIN/CD147 in regulating angiogenesis in patients with psoriatic arthritis

**DOI:** 10.1186/s13075-020-02333-6

**Published:** 2020-10-14

**Authors:** Michal A. Rahat, Mirna Safieh, Elina Simanovich, Eliran Pasand, Tal Gazitt, Amir Haddad, Muna Elias, Devy Zisman

**Affiliations:** 1 Immunotherapy Laboratory, Carmel Medical Center, 3436212 Haifa, Israel; 2 Ruth and Bruce Rappaport Faculty of Medicine, Technion-Israel Institute of Technology, 3109601 Haifa, Israel; 3 Rheumatology Unit Carmel Medical Center Haifa Israel, 3436212 Haifa, Israel

**Keywords:** Psoriatic arthritis (PsA), Angiogenesis, EMMPRIN/CD147, Thrombospondin-1 (Tsp-1)

## Abstract

**Background:**

Angiogenesis plays a central role in the pathophysiology of rheumatic diseases. Patients with psoriatic arthritis (PsA) demonstrate increased vascularity over patients with rheumatoid arthritis (RA), with unknown mechanisms.

**Methods:**

We evaluated the serum levels of several pro- and anti-angiogenic factors in 62 PsA patients with active disease, 39 PsA patients in remission, 33 active RA patients, and 33 healthy controls (HC). Additionally, we used an in vitro co-culture system of fibroblast (HT1080) and monocytic-like (MM6) cell lines, to evaluate how their interactions affect the secretion of angiogenic factors and angiogenesis promoting abilities using scratch and tube formation assays.

**Results:**

PsA patients, regardless of disease activity, exhibited higher levels of EMMPRIN/CD147, IL-17, and TNF-α relative to RA patients or HC. Factors, such as IL-6, and the ratio between CD147 and thrombospondin-1, exhibited elevated levels in active PsA patients relative to PsA patients in remission. Secretion of CD147, VEGF, and MMP-9 was increased in vitro. CD147 neutralization with an antibody reduced these levels and the ability of endothelial cells to form tube-like structures or participate in wound healing.

**Conclusions:**

CD147 plays a role in mediating angiogenesis in PsA, and the therapeutic possibilities of neutralizing it merit further investigation.

## Background

Angiogenesis, the process of sprouting of the new blood vessels from existing ones, is a complex process that requires balance between many pro- and anti-angiogenic factors. Although angiogenesis is mostly studied in the tumoral context, it is also demonstrated in rheumatic diseases to different degrees and has been implicated in their pathophysiology [1]. The increased immune infiltrate demands more oxygen than is locally available, resulting in hypoxia [2], which is a potent inducer of angiogenesis. Thus, angiogenesis supports the inflammatory process, and these two processes are inseparable. Interestingly, patients with psoriatic arthritis (PsA) tend to show increased vascularity and higher levels of synovial pro-angiogenic factors than patients with rheumatoid arthritis (RA) [3], and their blood vessels are more tortuous in comparison to RA patients that show straight and branching vasculature [4, 5]. However, the molecular mechanisms that explain these differences are not known.

Hypoxia increases the expression of many pro-angiogenic factors, such as vascular endothelial growth factor (VEGF) [6], which is also a strong chemoattractant for macrophages [7]. Macrophages and other infiltrating immune cells secrete enhanced levels of pro-inflammatory cytokines and chemokines in the inflamed synovium, such as interleukin-1β (IL-1β), IL-6, IL-8, IL-17, interferon β (IFN-β), monocyte chemoattractant protein 1 (MCP-1), and tumor necrosis factor α (TNF-α) that help recruit leukocytes to sites of inflammation [8]. VEGF and matrix metalloproteinases (MMPs) such as MMP-9 that are secreted by synovial tissue cells and immune infiltrating cells [5] activate endothelial cells to proliferate, migrate, and form tube-like structures with high permeability. IL-17 and TNF-α are crucial not only as pro-angiogenic factors, but also because they induce the expression of many MMPs that play a role in cartilage erosion and degradation [9, 10].

EMMRPIN/CD147 is a unique multifunctional protein, best known for its ability to regulate the expression of both VEGF and several MMPs via a homophilic interaction with another CD147 molecule [11–13]. CD147 is expressed on many cell types, including activated macrophages, T cells, fibroblasts, and endothelial cells, and has been shown to mediate the interactions between tumor epithelial cells and monocytes that are responsible for the induction of VEGF and MMP-9, even in its soluble form [14]. Thus, CD147 is an important pro-inflammatory and pro-angiogenic protein. CD147 has been studied mostly in the tumoral context, and only few studies have been published to this date demonstrating its elevated levels in RA and psoriasis patients [15–19]. Its role in PsA has not yet been investigated.

Our aims were to explore the angiogenic profile of PsA patients by delineating the serum expression of several pro- and anti-angiogenic key mediators, including CD147, and compare it to RA patients and healthy controls. We also investigated the role that CD147 plays in the regulation of angiogenesis and the results of its disruption using an in vitro co-culture system of fibroblasts and monocytes, as well as the wound scratch and tube formation assays.

## Methods

### Patients

The study cohorts included 101 PsA patients fulfilling the Classification Criteria for Psoriatic Arthritis (CASPAR) criteria [20], 33 RA patients who fulfilled ACR/EULAR 2010 criteria [21, 22], and 33 healthy controls (HC) without any inflammatory articular diseases who were matched for age and sex with the group of patients with inflammatory arthritis. All patients were followed in the Rheumatology Unit at Carmel Medical Center and were enrolled consecutively. The following parameters were collected: demographic data, age at disease onset, smoking and alcohol use, height, weight, and comorbidities. Data regarding current treatment with steroids and disease-modifying anti-rheumatic drugs (DMARDs), either conventional DMARDS (cDMARDS) (e.g., methotrexate, leflunomide, sulfasalazine, hydroxychloroquine) or biologic DMARDs (bDMARDS) (i.e., TNF-*α* inhibitors such as etanercept, infliximab, adalimumab, golimumab, abatacept, tocilizumab, rituximab, secukinumab, and ustekinumab). Serum samples were obtained from the participants and stored at − 80 °C for later analysis of the cytokines. The research was reviewed and approved by the local Institutional Review Board at Carmel Medical Center (CMC-004411), and all participants signed informed consent before enrollment.

### Sandwich enzyme-linked immunosorbent assay (ELISA)

Concentrations of CD147, VEGF, MMP-9, TNF*-α*, TGF-*β*, IL-6, IL-17, endostatin, and thrombospondin-1 (Tsp-1) were measured using commercial DuoSet ELISA kits (R&D systems, Minneapolis, MN) according to the manufacturer’s instructions. According to preliminary calibration experiments, duplicate serum and supernatant samples were diluted 1:100 in all kits, except for the endostatin kit (1:200) and the thrombospondin-1 kit (1:1000).

### Cells

The human fibrobsarcoma cell line HT1080 (ATCC CCL-12012) was cultured in Dulbecco’s modified Eagle’s medium (DMEM, Biological Industries, IL), 10% fetal calf serum (FCS), l-glutamine (2 mM), amphotericin B (27 μM), non-essential amino acids (NEAA, 0.1 mM each of alanine, aspargine, spartic acid, glutamic acid, glycine, proline, and serine), and 1% antibiotics (penicillin 0.016 mM, streptomycin 0.15 mM, neomycin 0.11 mM), with addition of 25% conditioned medium supplement obtained from the human promyelocytic leukemia cells HL60 (ATCC CRL-240) that contains secreted fibroblast growth factor-2 (FGF-2). The human monocytic-like cell line MonoMac6 (MM6, ATCC 25177) was cultured in RPMI-1640 medium, 10% FCS, l-glutamine (2 mM), amphotericin B (27 μM), NEAA (0.1 mM each), sodium pyruvate (100 mM), and insulin (10 μg/mL). The human endothelial cell line EaHy926 (ATCC CRL-2922) was cultured in DMEM with 10% FCS, glutamine (2 mM), 2% HAT (hypoxanthine 0.1 mM, aminopterin 0.4 μm, thymidine 16 *μ*m), and 1% antibiotics. Before the beginning of the experiments, the medium was replaced with serum-free medium with 0.1% BSA. All cell lines were split twice a week at a ratio of 1:4. To avoid masking of signals, cells were seeded in plates, and following cell adherence, medium was replaced with serum-starved medium (the HT1080 medium without FCS and with 0.1% BSA) for the duration of the experiment. All cell lines were regularly tested for morphological changes and presence of mycoplasma.

HT1080 (4 × 10^5^ cells) or MM6 (4 × 10^5^ cells) were cultured alone or in co-culture, in the absence or presence of TNF-*α* (1 ng/mL). Supernatants were collected after 48 h of incubation for further analysis. In some experiments, recombinant CD147 (R&D systems, Minneapolis, MN) in the doses indicated or anti-CD147 neutralizing antibody (2 ng/ml, BioLegend, San Diego, CA) were added to some of the wells.

### In vitro wound scratch assay

The assay was carried out using the EaHy926 endothelial cells, which were seeded (10^5^ cells/well) in 96-well plates and incubated with supernatants derived from experiments where HT1080 and MM6 cells were co-cultured with or without the addition of anti-CD147 antibody (2 ng/mL, BioLegend, San Diego, CA). To simulate injury, the endothelial confluent layer was scratched using a toothpick, and the non-adherent cells were washed away before the addition of the supernatants. Images of the field of the scratch were acquired at the beginning of the experiment (T0) and after 24 h (T24) (Moticam 2MP, × 4 magnification), and the wound area was measured at both times using the ImagePro plus 4.5 software (Media Cybernetics, Inc., Rockville, MD, USA). The migration area, reflecting the area to which endothelial cells migrated to close the wound, was calculated by the subtraction of the area at T24 from the area at T0.

### In vitro tube formation assay

The Coultrex® reduced growth factor basement membrane extract (40 μl/well, Trevigen, Gaithersburg, MD, USA) was used to coat 96-well plates at 4 °C and then was incubated at 37 °C for 2 h to allow it to polymerize. EaHy926 cells (8 × 10^4^ cells/well) were seeded in triplicates in DMEM with 2% FCS, and the experimental supernatants were diluted 1:2 with medium. Images of the wells were captured after 6 h of incubation (Moticam 2MP, magnification × 4) and the closed lumens, representing two-dimensional tube-like structures, were counted in two separate fields.

### Statistics

All values are presented as means ± standard error of measurement (SEM). Heterogeneity between study groups was calculated using the Fisher’s exact test. The nonparametric Kruskal-Wallis analysis of variance (ANOVA) test was used to compare multiple groups, followed by the Dunn’s multiple post hoc comparison test. To compare two groups, we used the two-tailed Mann-Whitney *U* test. Correlations between two variables were evaluated using the non-parametric Spearman test. *P* values exceeding 0.05 were not considered significant.

## Results

### Study population

The PsA group consisted of 101 patients with a median age of 60 years, of which 67 (65.6%) were female. Their demographics, comorbidities, and treatment regimen are reported in Table 1. Disease activity for PsA patients was determined by the minimal disease activity (MDA) score [23] and the DAPSA score [24]: 39 patients were classified in remission (MDA score ≥ 5), or remission (22 patients) and low disease activity (17 patients) according to the DAPSA score. In this group, the mean tender joint (TJ) count was 0.21 ± 0.08, swolen joint (SJ) count 0.15 ± 0.06, PASI score 1.5 ± 0.22, mean enthesial score 0.33 ± 0.12, and mean C-reactive protein (CRP) was 0.95 ± 0.41 mg/dL. In the active PsA group, TJ count was 10.30 ± 1.6, SJ count 7.03 ± 0.7, PASI score 3.9 ± 0.8, mean enthesial score 4.72 ± 0.72, and mean CRP was 4.7 ± 2.1 mg/dL. The RA group consisted of 33 patients, 78.8% of whom were females with rheumatoid factor (RF) positivity in 15 (45.5%). All had active disease as assessed by Clinical Disease Activity Index (CDAI) score > 11 [25] with a mean TJ of 14.5 ± 0.9, mean SJ of 10.90 ± 0.8, and mean CRP of 8.9 ± 1.2 mg/dL.

**Table 1:** Demographic characteristics, underlying diseases, and treatment of the study groups

	Active PsA	Remission PsA	Active RA	Healthy controls	***P*** values, active PsA vs.:	
					Remission PsA	Active RA
No. participants	62	39	33	33	–	–
Gender: female (%)	41 (66.1%)	26 (64.1%)	26 (78.8%)	26 (78.8%)	ns^3^	ns
Ethnicity: Jewish	52 (83.9%)	39 (100%)	25 (75.8%)	30 (90.1%)	0.0063	ns
Age (years)	56.6 ± 1.8	59.13 ± 2.1	57.8 ± 1.8	58.5 ± 1.7	ns	ns
Body mass index (BMI)	31.26 ± 1.1	26.3 ± 0.7	29.65 ± 1.1	26.9 ± 0.8	0.0027	ns
Disease duration: arthritis	12.84 ± 1.4	14.1 ± 1.88	6.93 ± 0.96	–	ns	0.0079
Disease duration: psoriasis	22.79 ± 1.8	36.59 ± 2.7	–	–	< 0.0001	–
Tobacco use (%)	27 (43.5%)	5 (13.2%)	13 (39.4%)	4 (13.3%)	0.0018	ns
**Comorbidities**						
Hypertension	21 (33.9%)	11 (28.2%)	11 (33.3%)	7 (21.2%)	ns	ns
Hyperlipidemia	27 (43.5%)	11 (28.2%)	18 (54.5%)	9 (27.3%)	ns	ns
Diabetes mellitus	16 (25.8%)	6 (15.4%)	9 (27.3%)	5 (15.2%)	ns	ns
Inflammatory bowel disease (IBD)	2 (3.22%)	0 (0%)	0 (0%)	–	ns	ns
Thyroid disease	5 (8.06%)	2 (5.1%)	4 (12.1%)	1 (3.03%)	ns	ns
Ischemic heart disease (IHD)	8 (12.9%)	0 (0%)	0 (0%)	2 (6.1%)	0.0194	0.0311
Cerebrovascular accident (CVA)	2 (3.2%)	0 (0%)	0 (0%)	–	ns	ns
**Medications**						
None			8 (24.2%)	–		
cDMARDs^1^ (total)	25 (40.3%)	23 (58.9%)	23 (69.7%)	–	ns	0.0094
Methotrexate	23 (37.1%)	17 (43.6%)	20 (60.6%)	–	ns	0.038
Leflunomide	4 (6.5%)	1 (2.6%)	7 (21.2%)	–	ns	0.0448
Sulfasalazine	6 (9.7%)	7 (17.9%)	–	–	ns	–
Hydroxychloroquine	1 (1.6%)	0 (0%)	7 (21.2%)	–	ns	0.0023
Glucocorticostreroids	2 (3.2%)	0 (0%)	15 (45.4%)	–	ns	< 0.0001
bDMARDs^2^ (total)	24 (38.7%)	17 (43.59%)	1 (3.0%)	–	ns	< 0.0001
Anti-TNFα agents (total)	23 (37.1%)	16 (41.0%)		–	ns	0.0001
Etanercept	11 (17.7%)	7 (17.9%)		–	ns	–
Golimumab	1 (1.61%)	2 (5.13%)		–	ns	–
Adalimumab	8 (12.9%)	4 (10.2%)		–	ns	–
Infliximab	3 (4.83%)	3 (7.7%)		–	ns	–
Abatacept			1 (3%)			–
Secukinumab	2 (3.22%)	0 (0%)	–	–	ns	–
Ustekinumab	3 (4.83%)	1 (2.56%)	–	–	ns	–

^1^*cDMARDs* conventional disease-modifying anti-rheumatic drugs

^2^*bDMARDs* biologic disease-modifying anti-rheumatic drugs

^3^*ns* not significant

### Disease activity affects serum levels of pro- and anti-angiogenic mediators in PsA patients

The levels of IL-17, TNF-*α*, and TGF-*β* were significantly increased in the active PsA group relative to patients with RA and HC (Supp. Figure 1a-d). Levels of TNF-*α* were increased in the two PsA groups relative to the active RA and the control groups, but without a statistically significant difference between them (Supp. Figure 1b).

**Fig. 1:**
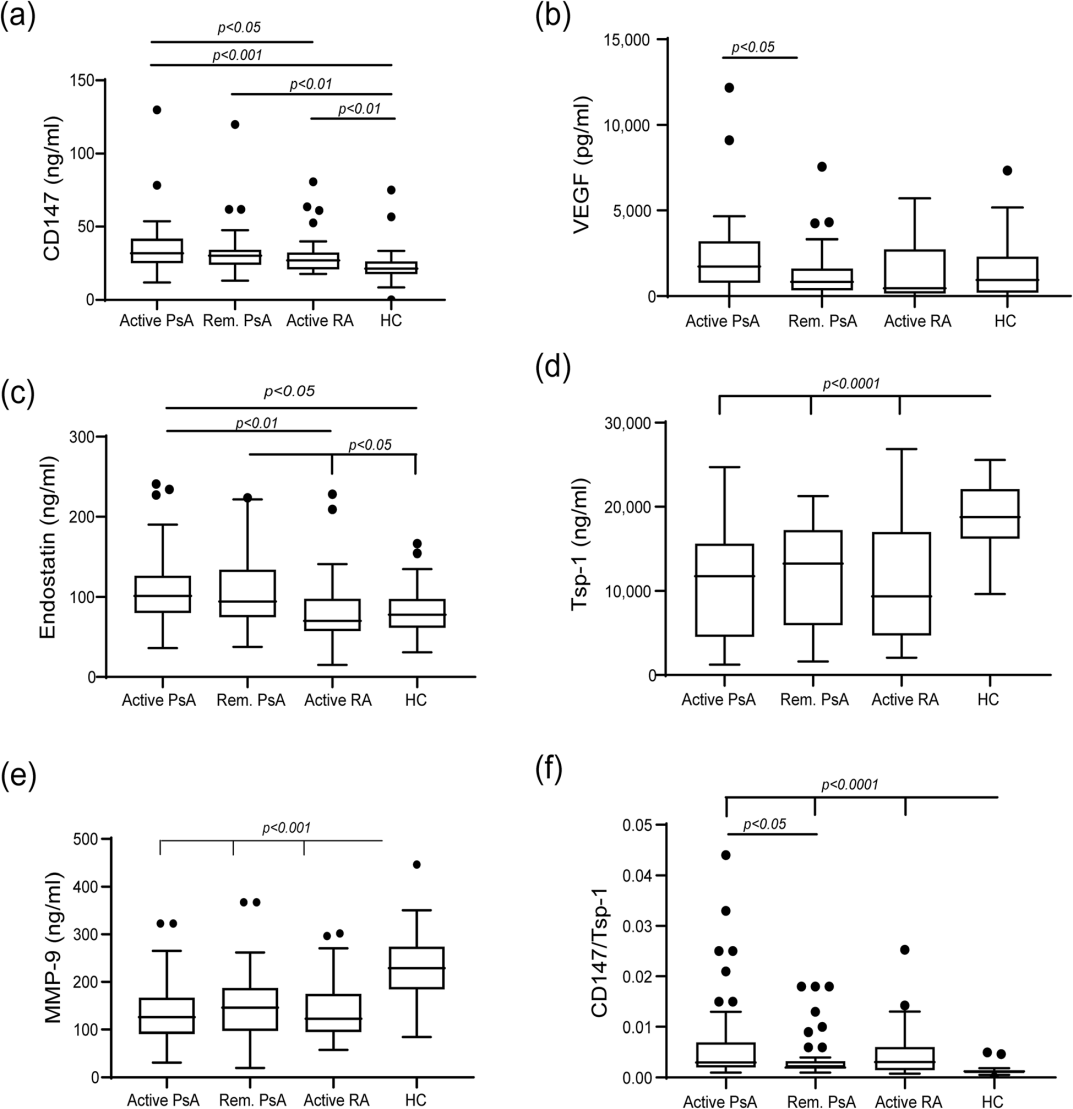
Serum concentrations of angiogenic factors: box plot representing the concentrations of angiogenic mediators in the serum of PsA patients with active disease (active PsA), PsA patients in remission (Rem. PsA), RA patients with active disease (active RA) and healthy volunteers (HC). **a** CD147, **b** VEGF, **c** endostatin, **d** thrombospondin-1 (Tsp-1), **e** MMP-9, and **f** the ratio between CD147 and Tsp-1 as a suggested measure of enhanced angiogenesis

The levels of the three major pro-angiogenic factors, CD147VEGF, and MMP-9, and two of the key anti-angiogenic factors, endostatin, and thrombospondin-1 (Tsp-1) were evaluated. Both CD147and endostatin were elevated in PsA patients relative to RA patients or to HC, with no significant difference between active PsA patients and patients in remission (Fig. 1a, c). CD147 levels, but not those of endostatin, were higher in the RA group than in the HC group. In contrast, VEGF serum levels were decreased when PsA patients were in remission relative to the active PsA group, but no difference was observed between the active PsA, active RA, and HC groups (Fig. 1b). Serum levels of MMP-9 were decreased in both the PsA groups and in the active RA group relative to the HC, with no significant difference between them (Fig. 1e). The anti-angiogenic factor Tsp-1 exhibited, as expected, a reverse profile, where the HC demonstrated the highest levels relative to all other patient groups (Fig. 1d). Since angiogensis is enhanced when the balance between pro- and anti-angiogenic factors favors the former, we calculated for each patient the ratio between the levels of the pro-angiogenic CD147and the anti-angiogenic Tsp-1 (Fig. 1f). The results show that both active PsA and active RA patients demonstrated a higher ratio than that of PsA patients in remission or the HC, strengthening the results obtained from each factor alone. In contrast, the ratio between VEGF and Tsp-1 did not improve on the already clear responses exhibited by each of those mediators alone (data not shown).

To acertain that the ratio between the pro-angiogenic factor CD147 and the anti-angiogenic factor Tsp-1 may describe the angiogeic status of PsA patients, we correlated this ratio to the serum levels of the factors tested in PsA patients (both patients with active disease and those in remission) as well as HC (Table 2). The ratio correlated significantly with the DAPSA and MDA clinical scores and with IL-6, and negatively correlated with Tsp-1 and MMP-9.

**Table 2:** Correlations of CD147/TSP-1 ratio to the serum levels of various factors in PsA patients and healthy controls (HC)

	Spearman ***r***	95% CI	***P*** value
CD147	0.628	0.509 to 0.723	< 0.0001
Tsp-1	− 0.845	− 0.888 to − 0.786	< 0.0001
VEGF	0.102	− 0.074 to 0.271	ns
MMP-9	− 0.516	− 0.633 to − 0.375	< 0.0001
Endostatin	0.278	0.109 to 0.431	0.0012
DAPSA	0.492	0.347 to 0.614	< 0.0001
PASI	0.362	0.200 to 0.505	< 0.0001
MDA	0.342	0.178 to 0.488	< 0.0001
IL-6	0.336	0.171 to 0.482	< 0.0001
TGF-β	− 0.103	− 0.300 to 0.104	ns
TNF-α	0.190	0.016 to 0.353	0.0281
IL-17	0.174	− 0.0007 to 0.338	0.0446

### Co-culture enhances the expression of CD147, MMP-9, and VEGF

An in vitro system that simulates cellular interactions between the two key cell types—macrophages (the monocytic cell line MonoMac6-MM6) and fibroblasts (the fibroblast cell line HT1080)—was used to explore our findings further. Previous results in our laboratory demonstrated the need for the presence of TNF*-α* to induce MMP-9, and an incubation period of 48 h was required to allow for the sufficient accumulation of factors in the supernatants.

**Fig. 2:**
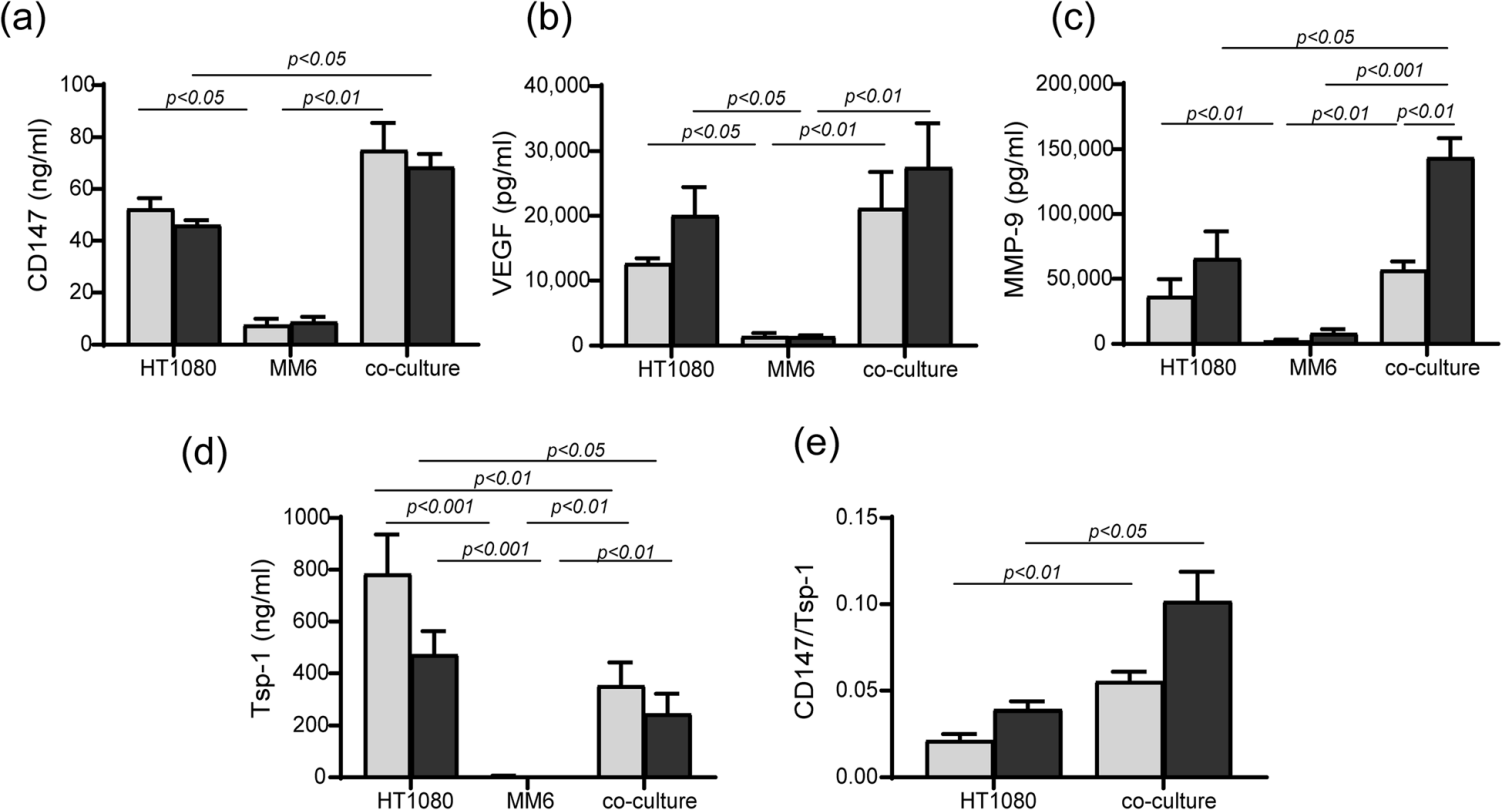
Co-culture enhances the secretion of some pro-angiogenic factors. HT1080 (4 × 10^5^ cells) were co-cultured with either MM6 (4 × 10^5^ cells), with and without the addition of TNF-α (1 ng/mL) and compared to each single culture. Supernatants were collected after 48 h of incubation and the concentrations of **a** CD147 (*n* = 6), **b** VEGF (*n* = 6), **c** MMP-9 (*n* = 6), and **d** Tsp-1 (*n* = 6) were determined by ELISA. **e** The CD147/Tsp-1 ratio was calculated for each repetition

We show that the monocytic cell line alone did not produce significant levels of *CD147*, VEGF, MMP-9, or Tsp-1, even in the presence of TNF-α (Fig. 2a–d). Addition of TNF-α was indeed necessary to increase MMP-9 accumulation but had no effect on any of the other factors tested (Fig. 2a–d). Most importantly, the co-culturing of the fibroblasts with monocytes in the presence of TNF-α increased *CD147* and MMP-9 levels, but not those of VEGF (Fig. 2a–d), whereas Tsp-1 levels were reduced (Fig. 2d). As observed in the patients’ serum samples, the ratio *CD147*/Tsp-1 significantly increased in the co-culture relative to the fibroblasts alone (Fig. 2e).

### CD147mediates the interactions between fibroblasts and monocytes

**Fig. 3:**
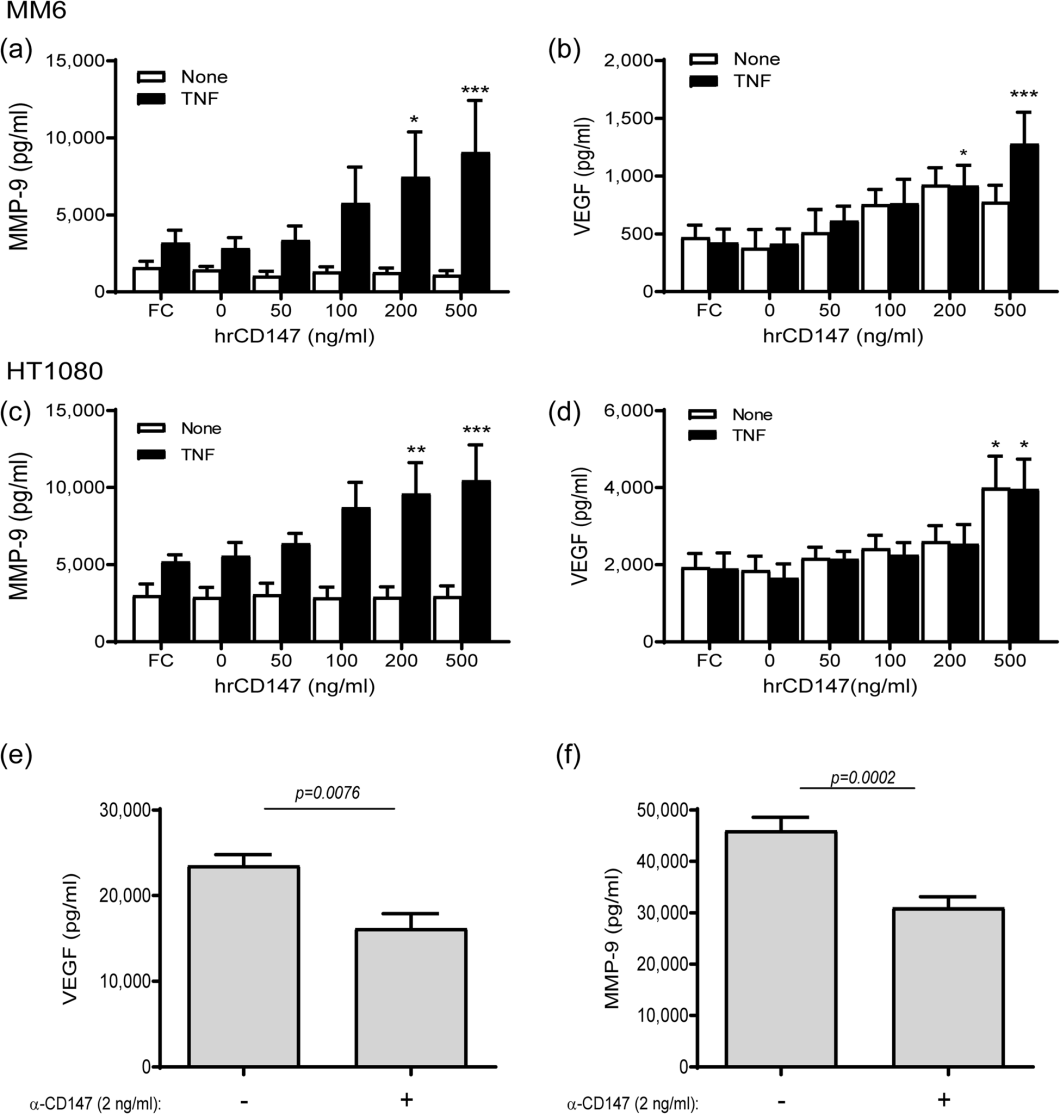
CD147mediates the increase in MMP-9 and VEGF. The HT1080 and MM6 cell lines (2 × 10^4^ cells) were cultured, with or without TNF*-α* (1 ng/mL), and the IgG Fc fragment (Fc, at 200 ng/ml) or increasing concentrations of recombinant human CD147 were added as indicated. After 48 h of incubation, supernatants were collected and concentrations of **a**, **c** MMP-9 and **b**, **d** VEGF were determined by ELISA (*n* = 6–7 in all groups). *p < 0.05, **p < 0.01, and ***p < 0.001 compared to cells with TNF-*α* and without recombinant CD147. Alternatively, HT1080 and MM6 cells (2 × 10^4^ each) were co-cultured in the presence of TNF-*α* (1 ng/mL), and with or without the addition of a neutralizing anti-CD147antibody (2 ng/mL). After 48 h of incubation, supernatants were collected and evaluated for their concentrations of **e** VEGF and **f** MMP-9 using ELISA (*n* = 8 in each group)

The increase in CD147, VEGF, and MMP-9 in the co-cultured fibroblasts and monocytes suggests that CD147, known to induce VEGF and MMPs, was responsible for mediating the interactions between these two cell lines. To show this, we incubated each cell type alone with increasing amounts of human recombinant CD147, with or without TNF-*α*. Both cell lines demonstrated that the presence of TNF-*α* was necessary to induce MMP-9, but had no influence on the accumulation of VEGF (Fig. 3a–d). Both VEGF and MMP-9 showed a clear dose-response to recombinant CD147, where the highest concentration used (500 ng/mL) resulted in a significant increase in their levels relative to the untreated control. Conversely, we co-cultured the two cell types in the presence of TNF-*α* adding anti-CD147antibody to the supernatants and showed that both VEGF and MMP-9 levels decreased in response to this treatment (Fig. 3e, f).

### CD147 mediates the angiogenic potential of the fibroblast-monocyte co-culture

**Fig. 4:**
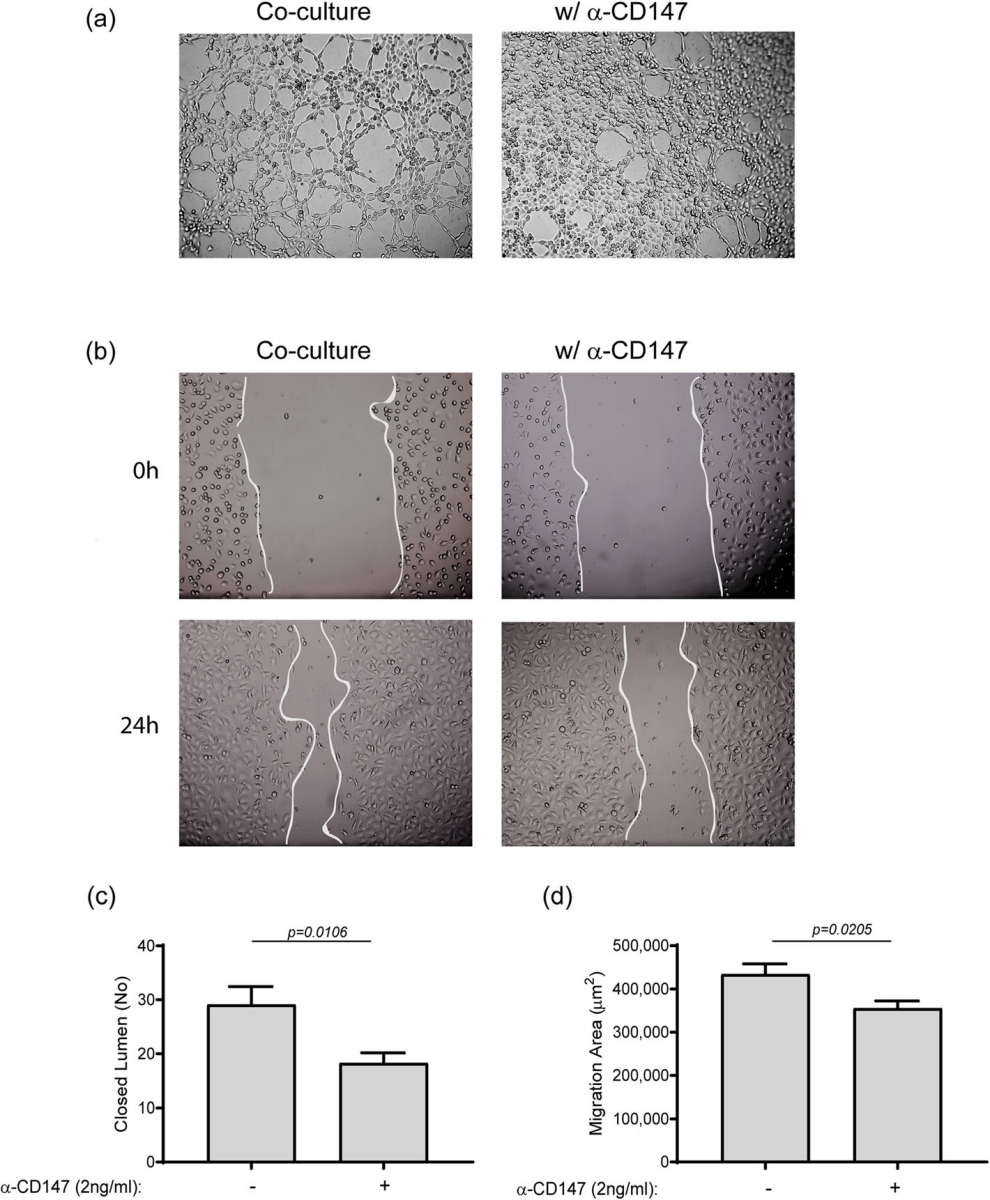
Co-culture of fibroblasts and monocytes enhances angiogenic potential, mediated through CD147. Supernatants from co-cultures of HT1080 and MM6 cells derived from the previous experiments were diluted 1:2 in full medium, with or without the neutralizing anti-CD147antibody (2 ng/mL), and incubated for 6 h with the human EaHy926 endothelial cells (8 × 10^4^ cells) which were seeded in wells coated with Coultrex®. **a** Representative images (×20 magnification) and **c** quantification of the closed lumen tube-like structures (*n* = 10). Confluent EaHy926 endothelial cells were scratched and washed, and **b** representative images obtained at the beginning of the experiment (0 h) and after 24 h (×20 magnification). **d** The subtraction of the area at T24 from the area at T0, resulting in the area to which endothelial cells migrated to close the wound migration area, was calculated (*n* = 7)

To examine the full angiogenic potential of the interaction between the two cell types, and explore the angiogenic role of CD147, we incubated the human endothelial cell line EaHy926 with the diluted supernatants derived from the co-culture experiments, with or without the presence of anti-CD147antibody. The tube formation assay demonstrated that the co-culture supernatants stimulated formation of many tube-like structures, whereas the addition of the antibody reduced that the number and increased the thickness or number of endothelial layers between the tubes (Fig. 4a, c). The wound scratch assay demonstrated an enhanced migration/proliferation rate in the co-cultured supernatants that was reduced upon the addition of the antibody (Fig. 4b, d).

## Discussion

Angiogenesis is an important component in the pathophysiology of rheumatic diseases, but although it has been described to be more enhanced in PsA than in RA patients [3], the molecular mechanisms that regulate this phenomenon are still unclear. Many pro-angiogenic factors have been associated with PsA, but those were mostly studied in the synovial tissue or the synovial fluid. Here, we evaluated the serum concentrations of pro- and anti-angiogenic factors and demonstrated that the balance between them in active PsA patients, more than in the RA or HC, has shifted toward the pro-angiogenic factors. Specifically, we show that PsA patients with active disease exhibit higher serum levels of CD147, relative to RA patients or HC. Using an in vitro co-culture system, we show that CD147 mediates the interactions between fibroblasts and monocytes and enhances angiogenesis through the increased expression of VEGF and MMP-9, placing it as an important, but not sole mediator of PsA pathophysiology.

Our results showing enhanced cytokine levels in serum from active PsA patients (Fig. S1) are consistent with previous studies that have shown a central role for IL-17, IL-6, TGF-**β**, and TNF-**α** in PsA patients [8]. Since these inflammatory cytokines also have an important pro-angiogenic role [8], their enhanced serum levels in active PsA patients further supports the presence of enhanced angiogenesis reported in PsA patients.

CD147 is an important pro-angiogenic factor, as it can participate in the regulation of both VEGF and MMPs. Its enhanced secretion by monocytes derived from RA patients was previously described [15]. Here, we demonstrate elevated CD147 levels in serum obtained from active RA patients relative to HC, and even higher levels in serum samples obtained from PsA patients regardless of disease activity. This may reflect the enhanced angiogenesis observed in PsA patients reflected by the formation of long, tortuous, and dilated vessels in the synovium, in comparison to RA patients who have thickened, straight, and evenly branched vessels in the synovial tissue [8, 26]. This suggests that CD147 plays a role in rheumatic diseases in general, and in PsA in particular, in accordance with the important role of angiogenesis in the pathogenesis of PsA. This is also supported by similar studies demonstrating elevated serum levels of CD147 in patients with psoriasis [27] and elevated angiogenesis and expression of CD147 in fibroblast-like synoviocytes (FLS) [28]. The important role played by CD147 in angiogenesis is also highlighted by our in vitro studies, which emphasize the need for interaction between at least two cell types to enhance CD147 concentrations and its ability to induce the potent pro-angiogenic factors VEGF and MMP-9. Moreover, in view of the role CD147 plays in chemotaxis and Th17 cell differentiation in lymphocytes or neutrophils derived from RA patients [19, 29], its targeting by an antibody might prove beneficial in human PsA patients.

The elevated CD147 levels found in both PsA groups were not mirrored by these patients’ VEGF serum levels, which were similar to those seen in the active RA or HC groups. This may reflect the high variability of the VEGF assay or the suppressive effects of medications taken by the PsA patients in remission. The role played by additional regulatory factors [such as Hypoxia-Inducible Factor-1 (HIF-1), Signal Transducer and Activators of Transcription 3 (STAT3), Mitogen-Activated Protein Kinase (MAPK), and p53] in VEGF expression suggests a complex regulatory process involving this mediator in disease pathogenesis [30].

MMPs primarily degrade extra-cellular matrix (ECM) proteins, and as such, they play an important role in damage to the bone and cartilage characteristic of rheumatic diseases. Additionally, MMPs also regulate angiogenesis, as they facilitate endothelial cell migration and proliferation [10]. PsA and RA are both associated with elevated levels of MMP-9 and other MMPs [10]. Despite this, we demonstrated reduced levels of MMP-9 in all the patients relative to HC. Different drugs, such as methotrexate (MTX), sulfasalazine (SSZ), several statins, infliximab, and other TNF-α blockers, were shown to reduce serum levels of MMP-9, [15, 31–37]. Thus, the reduction in MMP-9 levels seen in the PsA and RA patient groups in our study may be partially explained by their baseline treatment. Of note, we conducted several experiments to rule out the possibility that the reduction in MMP-9 was due to the ELISA kit we used, a kit that recognizes pro- and active-MMP-9, but not MMP-9 bound to TIMP-1. We repeated the test with a kit that recognizes only the bound form of MMP-9 and used a fluorescently labeled substrate to evaluate MMP-9 activity, all with similar results to those we obtained with the initial kit (data not shown).

Both endostatin and Tsp-1 are known as potent anti-angiogenic factors, and we expected them both to show reduced levels in the inflammatory groups relative to HC, reflecting the enhanced angiogenic process found especially in PsA patients. To our surprise, endostatin exhibited elevated levels in PsA patients regardless of disease activity. These results are in agreement with a study demonstrating elevated serum levels of endostatin in patients with systemic sclerosis where elevated endostatin reflected the degree of vascular injury [38]. Since, to the best of our knowledge, our study is the first to measure serum levels of endostatin in PsA and RA patients, we can only assume that these elevated levels point to additional functions of endostatin, such as its ability to inhibit fibroblast proliferation or attenuate osteoclast formation [39, 40]. Likewise, Tsp-1 serum levels were also not previously studied in PsA patients, and conflicting data regarding Tsp-1 serum levels come from studies in other rheumatic diseases (e.g., RA, SLE) [41, 42]. Our results show decreased Tsp-1 levels in PsA and RA patients relative to HC and are in agreement with the decreased levels found in SLE patients [42], requiring further exploration.

Notably, the factors tested in this study are mediators not only of angiogenesis, but also of inflammation, cell proliferation, and leukocyte recruitment and activation [8]. Thus, it is difficult to separate their pro-angiogenic role from their other functions in vivo. Therefore, we turned to the in vitro co-culture system, where their angiogenic effect, and particularly the effect of CD147, could be studied. We show that in an in vitro co-culture of fibroblasts and monocytes, CD147 plays an important role in mediating interactions between these two cell types, as the addition of recombinant protein mimicking homophilic CD147 interactions induced secretion of VEGF and MMP-9, and CD147 inhibition with the anti-CD147 antibody lowered this secretion. Likewise, the inhibiting effect of the anti-CD147 antibody on the formation of tube-like structures and on the migration and proliferation of endothelial cells demonstrates that these interactions and their resulting increased VEGF and MMP-9 levels directly affect endothelial cells, the cell most intimately involved in angiogenesis.

The fact that CD147 inhibition did not result in complete inhibition of these factors suggests that other mediators may also affect VEGF and MMP-9 levels. The discrepancies between the in vivo influence and the in vitro systems (e.g., changes in the MMP-9 levels) may arise from the lack of influence from other cell types and remote organs (such as the inflicted skin and synovium) in the in vitro system.

Lastly, the pro-angiogenic state of a PsA patient may be expressed by calculating the ratio of CD147 and Tsp-1. We show here that CD147 is an important mediator of the interactions between fibroblasts and monocytes and that this interaction, in turn, leads to the induction of the potent pro-angiogenic factors VEGF and MMP-9, rendering it a pro-angiogenic protein by itself. In contrast to CD147, Tsp-1 is known to be a strong anti-angiogenic factor [43]. This ratio, which is elevated in active inflammation and reduced in disease remission, may reflect the state of angiogenesis in these conditions. This is supported by the significant correlations found between this parameter and other pro-angiogenic factors in this study. However, further studies are needed to demonstrate the relevance of this ratio in clinical practice.

Our study has some limitations. The small size of the patient cohorts, especially of the control groups, may have masked the significance of our findings in some instances. Our active PsA patients used tobacco more than the patients in remission, and this could enhance angiogenesis in this group [44]. Most of our PsA and RA patients were treated. We did not have access to fibroblast-like synoviocytes (FLS) or to synovial fluids, and therefore, we used the fibroblast cell line HT1080 in our in vitro experiments. Since the concentrations of many of the tested factors are regulated locally, a future study should verify our in vivo findings in co-cultures of FLSs and monocytes.

## Conclusions

We show here that a complex network of cytokines possessing both pro-inflammatory and pro-angiogenic functions plays a role in the immunopathogenesis of PsA. We emphasize the role played by CD147 in mediating the interactions between fibroblasts and monocytes and in promoting angiogenesis in PsA patients, and suggest that the potential benefit of anti-CD147 therapy in patients with PsA should be further investigated.

## Supplementary information

**Additional file 1: Figure S1.** Serum concentrations of pro-inflammatory and anti-inflammatory cytokines: box plot representing in the serum of PsA patients with active disease (Active PsA), PsA patients in remission (Rem. PsA), RA patients with active disease (Active RA) and healthy volunteers (HC) the concentrations of (a) IL-17, (b) TNF-α (c) IL-6, and (d) TGF-β, as evaluated by ELISA.

## Data Availability

All data generated or analyzed during this study are included in this published article and its supplementary information files.

## Abbreviations

CASPAR: Classification Criteria for Psoriatic Arthritis
bDMARDs: Biologic disease-modifying anti-rheumatic drugs
cDMARDs: Conventional disease-modifying anti-rheumatic drugs
DMARDs: Disease-modifying anti-rheumatic drugs
ELISA: Enzyme-linked immunosorbent assay
EMMPRIN/CD147: Extracellular matrix metalloproteinase inducer/cluster of differentiation 147
ERK: Extracellular receptor kinase
HC: Healthy controls
IFN: Interferon
IL: Interleukin
MAPK: Mitogen-activated protein kinases
MCP-1: Monocyte chemoattractant protein 1
MMP: Matrix metalloproteinase
NEAA: Non-essential amino acids
PsA: Psoriatic arthritis
RA: Rheumatoid arthritis
TGF-β: Transforming growth factor β
Tsp-1: Thrombospondin*-*1
TNF-α: Tumor necrosis factor α
VEGF: Vascular endothelial growth factor
