# Supplementary figures and images for: The role of EMMPRIN/CD147 in regulating angiogenesis in patients with psoriatic arthritis
Source: Arthritis Res Ther. 2020 Oct 14;22:240. doi: 10.1186/s13075-020-02333-6 (PMC7557017; doi:10.1186/s13075-020-02333-6)

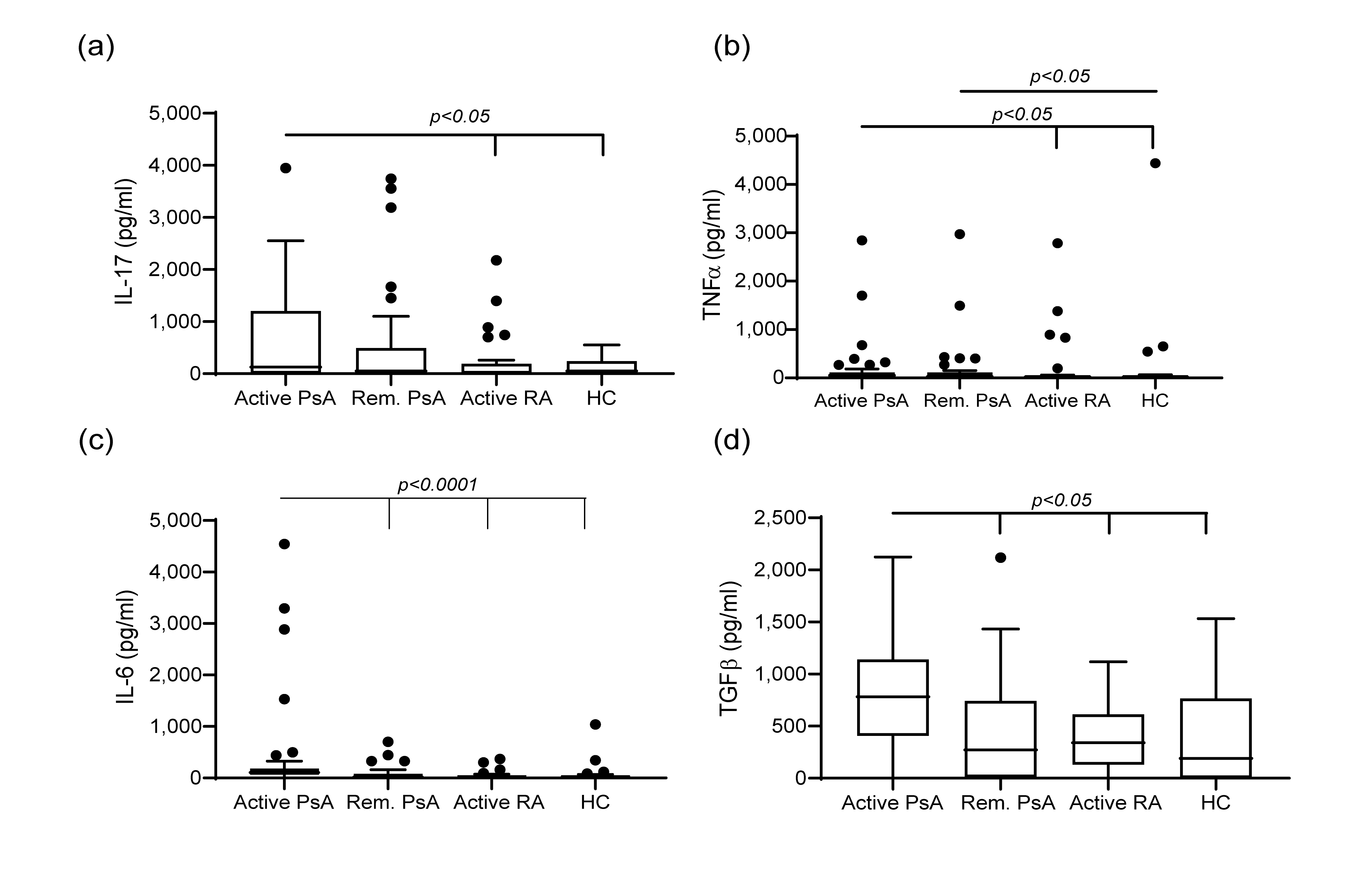

Supplement: Supplementary file 1 — Additional file 1: Figure S1. Serum concentrations of pro-inflammatory and anti-inflammatory cytokines: box plot representing in the serum of PsA patients with active disease (Active PsA), PsA patients in remission (Rem. PsA), RA patients with active disease (Active RA) and healthy volunteers (HC) the concentrations of (a) IL-17, (b) TNF-α (c) IL-6, and (d) TGF-β, as evaluated by ELISA. [file 13075_2020_2333_MOESM1_ESM.tif]
